# Cell therapy induced regeneration of severely atrophied mandibular bone in a clinical trial

**DOI:** 10.1186/s13287-018-0951-9

**Published:** 2018-08-09

**Authors:** Cecilie Gjerde, Kamal Mustafa, Sølve Hellem, Markus Rojewski, Harald Gjengedal, Mohammed Ahmed Yassin, Xin Feng, Siren Skaale, Trond Berge, Annika Rosen, Xie-Qi Shi, Aymen B. Ahmed, Bjørn Tore Gjertsen, Hubert Schrezenmeier, Pierre Layrolle

**Affiliations:** 1 Institute of Clinical Dentistry, University of Bergen, Bergen, Norway; 2 Institute of Transfusion Medicine, Ulm University, Ulm, Germany; 3 Institute for Clinical Transfusion Medicine and Immunogenetics Ulm, Red Cross Blood Service Baden-Württemberg—Hessen and Institute for Transfusion Medicine, University Hospital Ulm, Ulm, Germany; 4 Department of Fibre and Polymer Technology, KTH Royal Institute of Technology, 10044 Stockholm, Sweden; 5 Department of Internal Medicine, Hematology Section, Haukeland University Hospital, Bergen, Norway; 6 Centre for Cancer Biomakers CCBIO, Bergen, Norway; 7 Department of Clinical Science, Precision Oncology Research Group, University of Bergen, Bergen, Norway; 8 INSERM, UMR 1238, PHY-OS, Laboratory of Bone Sarcomas and Remodeling of Calcified Tissues, Faculty of Medicine, University of Nantes, Nantes, France

**Keywords:** Bone tissue engineering, Biphasic calcium phosphate, Dental implants, Alveolar ridge augmentation, Mesenchymal stem cells, Bone regeneration

## Abstract

**Background:**

Autologous grafting, despite some disadvantages, is still considered the gold standard for reconstruction of maxillofacial bone defects. The aim of this study was to evaluate bone regeneration using bone marrow-derived mesenchymal stromal cells (MSCs) in a clinical trial, a less invasive approach than autologous bone grafting. This comprehensive clinical trial included subjects with severe mandibular ridge resorption.

**Methods:**

The study included 11 subjects aged 52–79 years with severe mandibular ridge resorption. Bone marrow cells were aspirated from the posterior iliac crest and plastic adherent cells were expanded in culture medium containing human platelet lysate. The MSCs and biphasic calcium phosphate granules as scaffolds were inserted subperiosteally onto the resorbed alveolar ridge. After 4–6 months of healing, new bone formation was assessed clinically and radiographically, as were safety and feasibility. Bone at the implant site was biopsied for micro-computed topography and histological analyses and dental implants were placed in the newly regenerated bone. Functional outcomes and patient satisfaction were assessed after 12 months.

**Results:**

The bone marrow cells, expanded in vitro and inserted into the defect together with biphasic calcium phosphate granules, induced significant new bone formation. The regenerated bone volume was adequate for dental implant installation. Healing was uneventful, without adverse events. The patients were satisfied with the esthetic and functional outcomes. No side effects were observed.

**Conclusions:**

The results of this comprehensive clinical trial in human subjects confirm that MSCs can successfully induce significant formation of new bone, with no untoward sequelae. Hence, this novel augmentation procedure warrants further investigation and may form the basis of a valid treatment protocol, challenging the current gold standard.

**Trial registration:**

EudraCT, 2012-003139-50. Registered on 21 August 2013. ClinicalTrials.gov, NCT 02751125. Registered on 26 April 2016.

## Background

Bone is among the most frequently transplanted tissues, with about 2.2 million procedures annually worldwide [1]. In bone reconstruction procedures, surgeons harvest autologous bone from the patient and transplant this bone graft to the defect. This is currently regarded as the gold standard in bone regeneration, using the patient’s own cells and growth factors and providing scaffolding for bone regeneration [2]. However, the procedure has several major disadvantages: harvesting requires a second surgical site and provides only limited bone stock; the two-stage procedure increases surgery time; and patients often suffer pain and nerve damage at the harvest site. Moreover, autologous bone has an unpredictable resorption rate [3–5]. These factors all increase treatment costs and patient discomfort.

In the maxillofacial region, reconstruction may be necessary to treat congenital malformations, severe facial trauma, or resection of tumors [6, 7]. Bone defects also occur in the maxilla and mandible, often after tooth loss, which results in atrophy of hard and soft alveolar tissue, and reduction of both horizontal and vertical dimensions [2]. In clinical practice, patients often present with severe alveolar ridge resorption, leaving insufficient bone volume for optimal installation of dental implants. Various surgical procedures have been developed to enlarge the alveolar crest [8].

Because of the disadvantages inherent in autologous grafting, alternative methods for bone regeneration have been proposed, including bone substitutes of animal, human, or synthetic origin [9–12]. There are, however, documented cases of infection associated with bone substitute materials. Furthermore, the risks of bacterial contamination and immune rejection of the graft must be considered [9]. While these procedures can be used to reconstruct small bone defects, they are less effective in larger defects [3, 12, 13]. Thus, there is currently an unmet clinical need for effective, safe interventions which do not expose the patient to the risk of donor site morbidity [14–18].

Multipotent stromal cells or mesenchymal stem cells (MSCs) are the cells most extensively investigated and applied [19–30]. These cells are nonhematopoietic and of mesodermal derivation, capable of self-renewal and multilineage differentiation (e.g., into osteoblasts, adipocytes, and chondrocytes). MSCs are found throughout the body and numerous extraction protocols have been established for different tissues (e.g., umbilical cord, adipose tissue, skeletal muscle, deciduous teeth, and other tissue) [20, 21, 23, 24, 31, 32]. For more than 40 years, bone marrow-derived stem cells have been the most frequent sources for cell therapy. These cells can be isolated from bone marrow and from bone chips (cortical or trabecular bone). If seeded onto or cultivated on calcium phosphate ceramic matrices in vitro, these cells can induce bone formation in vivo [14, 33–35]. For many years, biphasic calcium phosphate (BCP) has been used alone or in combination with autologous bone chips to reconstruct the floor of the maxillary sinus and to fill extraction sockets [36, 37].

Recent preclinical studies have shown that BCP ceramics consisting of 20% hydroxyapatite (HA) and 80% beta tricalcium phosphate (β-TCP) are appropriate matrices for MSC culture in vitro and bone formation in vivo [38, 39]. In the present clinical study, the maxillofacial region was selected as an appropriate site for evaluating the safety and feasibility of using MSCs and BCP as a new therapeutic approach to regenerate alveolar bone defects. There were several reasons for this selection. Firstly, repair of facial bone defects is a major clinical challenge [40]. Currently, therapeutic options for repairing large, critical-sized defects are limited to autografts, allografts, or transplanting vascularized bone and soft tissue from autologous secondary sites [40]. Secondly, while a functional dentition is part of the normal facial anatomy, loss of teeth initiates a process of continuous resorption of the alveolar ridge. This is accelerated by denture wear and often results in pronounced loss of bone volume and reduction in the strength of residual bone in the edentulous area. Thirdly, reconstruction of the severely atrophic mandible to restore oral function remains a difficult surgical and prosthetic challenge because of the minimal residual bony volume and the progressive nature of the resorption process [41–43]. Although only a small proportion of edentulous people need bone augmentation for implant installation, for the patients who do, the procedure is essential for restoration of oral function and treatment options are limited [10]. Finally, the implant installation procedure makes it ethically acceptable to biopsy the implant site to inspect the quality of newly formed bone.

The present clinical trial in humans introduced a novel bone augmentation protocol. The primary aim was to introduce and validate the protocol, which uses bone marrow-derived MSCs for the clinical trial and synthetic BCP in a standardized, minimally invasive surgical procedure, and to assess the feasibility, safety, and efficacy of this new procedure. The autologous cells were harvested and cultured for 3 weeks before being implanted into the defect sites. The secondary outcome was to install dental implants in the augmented alveolar bone and screw-retain a fixed partial denture on the implants.

## Methods

### Ethical approval

This study conforms with the Declaration of Helsinki, and was approved by the Norwegian ethical committee (2013/1284/REK Vest, University of Bergen) and by the Norwegian Medicines Agency (13/12062-15; EudraCT 2012-003139-50). The clinical trial followed the European guidelines for advanced therapeutic medicinal products (ClinicalTrials.gov, NCT 02751125; https://clinicaltrials.gov/ct2/show/NCT02751125).

Two experienced clinicians informed the patients about the study. After signing the consent form, the patients underwent clinical examination, including clinical photographs and dental impressions, and provided a medical history. If the patient met the inclusion criteria, cone beam computer tomography (CBCT) (Morita 3D Accuitomo F17, Japan) and dental X-ray scans were taken.

### Study design and participants

Thirteen patients were recruited for this clinical study at the Section of Oral and Maxillofacial Surgery, Department of Clinical Dentistry, University of Bergen, Norway. To be eligible, the patients had to be healthy nonsmokers, with blood tests showing no evidence of infectious diseases, aged between 18 and 80 years, missing one or more teeth in the mandibular posterior region, and have an alveolar ridge width in the edentulous area less than 4.5 mm. All participants provided written informed consent before any study-related intervention. The study design and time points for each intervention are presented in Table 1.

**Table 1:** Study design and time schedule for the intervention

Intervention	Day –21 (pre inclusion visit or earlier)	Day 0 (inclusion visit)	Days 12–14	Month 1	Month 6	Month 9	Month 18
Verification of the selection criteria, information given to the patient, patient records and informed consent obtained	X						
Panoramic X-ray scans	X				X	X	X
Loco-regional clinical examination	X	X	X	X	X	X	X
Impression of both dental arches	X					X	
Facial and oral cavity photographs	X						X
Dental radiographs	X				X	X	X
VAS score for pain			X	X	X	X	X
Questionnaire on use of painkillers			X	X	X	X	X
Bone marrow harvest	X						
Grafting procedure		X					
CBCT scan	X		X		X		X
Implant placement, bone biopsy					X		
Resonance frequency Analysis (ISQ RFA)					X	X	X
Implant loading (prosthesis)						X	
Adverse events, clinical examination		X	X	X	X	X	X

*VAS* visual analog scale, *CBCT* cone beam computer tomography, *ISQ* implant stability quotient, *RFA* resonance frequency analysis

### Inclusion criteria

- Patients presenting with a subjective indication for a fixed implant-retained prosthesis in the mandibular posterior region (i.e., distal to the canine).
- Extensive lateral bone loss of the edentulous alveolar ridge.
- Edentulous alveolar ridge width less than 4.5 mm.
- Edentulous for more than 6 months in the region requiring reconstruction.
- At least one missing tooth to be replaced in the edentulous area.
- Absence of clinical signs of infection in the region requiring reconstruction.
- Absence of any major oral pathology.
- Age 18 years and older.
- In good health.

### Exclusion criteria

- Evidence of infection with HIV, or hepatitis B or C, or any contagious disease (specifically, serologically negative for anti-HIV 1–2 Ab, anti-HCV Ab, HBs Ag, anti-HBc syphilis, and negative (not detected by PCR) in HIV NAT, HCV NAT, or HBV NAT).
- Smoker.
- Pregnant or breastfeeding.
- Untreated infections.
- History of malignancy.
- History of or scheduled cervico-facial radiation therapy.
- Chronic treatment with steroids, immunomodulatory drugs, or bisphosphonates.

### Cell production

In 13 participants, bone marrow aspirates were harvested from the posterior iliac crest under local anesthesia at The Adult Clinical Trial Unit at Haukeland University Hospital, Bergen, Norway using a trocar to make two or three cutaneous punctures. Each bone marrow sample was harvested in fractions of 2–4 ml in 20-ml syringes prefilled with 1000 IU of heparin (Leo Pharma A/S, Denmark) and sealed with a Luer lock stopper (Omnifix 20 ml Luer Lock Solo; B. Braun Melsungen AG, Melsungen, Germany). A total of 15–20 ml of bone marrow aspirate from each patient was transported at 21 ± 3 °C with temperature recording and monitoring to provide traceability, and dispatched by a special courier service to the cell manufacturing center at the Institute for Clinical Transfusion Medicine and Immunogenetics (IKT), Ulm, Germany. This center has a production license for MSCs from BM aspirates (production license DE_BW_01_MIA_2013_0040/DE_BW_01_IKT Ulm), using Good Manufacturing Practices (GMP), according to defined standard operating procedures and in compliance with the established quality management system. The advanced therapy medicinal product MSCs were manufactured at IKT Ulm as previously described by Fekete et al. [44].

On arrival in Ulm, BM aspirates from the syringes were pooled and a cell count of the bone marrow was performed using an automated hematology analyzer (Sysmex KX-21 N; Sysmex Deutschland GmbH, Norderstedt, Germany) before any manipulation. Viability was evaluated by flow cytometry following 7-amino-actinomycin D staining (FC500 flow cytometer; Beckman Coulter, USA). If the total white blood cell (WBC) count was less than 127.2 × 10^6^ cells, the sample was considered inadequate for processing. Viability of MSCs (passage 0 and passage 1) was evaluated by Trypan blue staining (Sigma, Taufkirchen, Germany). All manipulations were conducted under laminar hood flow in grade A clean room conditions. The expansion was carried out as previously described [44]. In brief, the cell expansion started with 15–20 ml of bone marrow aspirate; the cells were seeded on one to eight 2-chamber CellSTACKs (Corning/Fisher Scientific, Schwerte, Germany) at a density of 50,000 WBCs/cm^2^ in Minimal Essential Medium alpha modification (αMEM) (Lonza, Basel, Switzerland), supplemented with 5% human platelet lysate (PL; IKT Ulm) and 1 IU/ml heparin (Ratiopharm, Ulm, Germany) for 14 days. The cells were then detached using trypsin (TrypZEAN; Lonza). The harvested passage 0 cells (MSC-P0) were counted and reseeded on one to seven 2-chamber CellSTACKs at a density of 4000 MSC-P0/cm^2^ in αMEM supplemented with 8% human platelet lysate and 1 IU/ml heparin (Ratiopharm) for 7 days. The cells were detached and passage 1 MSCs were washed with phosphate buffered saline without Ca^2+^/Mg^2+^ (Lonza), resuspended in a concentration of 20 × 10^6^ MSCs/ml in clinical-grade physiological saline (Kochsalz 0.9% INJ.-FL.(injection fluid), 50 ml; B. Braun Melsungen AG) supplemented with 4–5% human serum albumin (CSL Behring, Munich, Germany). Doses of 5 ml were drawn into one or two sterile syringes sealed with a Luer lock stopper. Transport was undertaken by a certified shipping company (World Courier, Stuttgart, Germany) as an accompanied transport to the clinical unit at the Department of Oral and Maxillofacial Surgery, Institute of Clinical Dentistry, University of Bergen, within 24 h of production. Appropriate quality controls of the cell therapy product were conducted after each step of the culture procedure. Viability and the number of cells were conducted using a Trypan blue viability test and a Countess Automated Cell Counter (Countess™; Invitrogen, Life Technologies, USA) respectively. Details on manufacturing the MSCs including quality controls are presented in a separate manuscript (Rojewski et al., submitted).

### Clinical procedures

All procedures were carried out under local anesthesia by an experienced surgeon (CG). CBCT scans were taken for each patient using a CBCT scanner (Morita 3D Accuitomo F17, Japan) to evaluate the bone volume before (T0) and 4–6 months after grafting (T1). One hour preoperatively, the patients received 1 g amoxicillin orally (or 300 mg clindamycin if allergic to penicillin). The site was surgically prepared under local anesthesia (Xylocain/adrenalin 2%; Astra Zenical AS, Sweden). A flap was raised and the cortical bone was then perforated with a small round burr, to enhance blood flow and facilitate vascular ingrowth into the biomaterials (Fig. 2A). Titanium-reinforced, nonresorbable polytetrafluoroethylene (PTFE) (Cytoplast; Osteogenics Biomedical, Lubbock, TX, USA) membranes were then fixed to the underlying bone by micro-screws and mini-screws (Biomet, Jacksonville, FL, USA) to provide a “tenting” effect [45–47].

For each patient, 5 cm^3^ of BCP (MBCP^+^™; Biomatlante, France), comprising 20% HA and 80% β-TCP in the form of granules 0.5–1 mm in size and packed in two syringes, were used and mixed with 100 million MSCs at the time of surgery. During this step, MSCs attached to the BCP granules in the syringes within a contact time of 60 min. The final number of cells mixed with BCP was in a dose of 20 × 10^6^ cells/1 cm^3^ [39]. When the graft was ready to be inserted, the BCP granules loaded with MSCs were withdrawn from the syringe and immediately inserted into the implant site (Fig.2B). Part of the mixture was preserved for additional analyses, particularly bacteriological tests and cell attachment on BCP. For cell attachment, the fluorescent dye DAPI (Sigma-Aldrich), which binds selectively to DNA and forms strongly fluorescent DNA–DAPI complexes, was used. The cell-seeded material was introduced into the pocket formed by the bony ridge and the regenerative membrane and then covered by the membrane and muco-periosteal flaps (Fig. 2C). Finally, the flaps were sutured to the vestibular mucosa using nonabsorbable sutures (4/0 Supramide; B. Braun Surgical SA, Spain).

The patients were instructed to eat only soft food for the next 10–14 days, and to rinse daily with chlorhexidine. The antibiotics were continued for 7 days. If necessary, pain was managed by oral administration of paracetamol (1 g tablets) or codeine phosphate sesquihydrate (30 mg) four times per day.

The operation site was examined clinically and the sutures were removed 12 days after surgery. CBCT scans were taken of the augmented area (T1). The patients were recalled for clinical examination after 1, 2, and 4 months (Fig. 2D). CBCT scans were taken 4–6 months postoperatively to determine whether the sites were ready for implant installation.

At the time of implant installation the augmented area was reentered if the width was 7 mm or more (Fig. 2E). Prior to implant installation, bone biopsies were taken under local anesthesia: new bone formation was assessed by histology and micro-computed tomography (μ-CT) (Skyscan 1172; Bruker) at 40 kV and 2.4-μm voxel size. Dental implants (Bone Level, Roxolid®, SLActive®; Institut Straumann AG, Basel, Switzerland) with a diameter of 4.1 mm and a length of 8–10 mm were then installed according to the manufacturer’s recommendations (Fig. 2F). Abutment surgery was done 2 months after implant installation (Fig. 2G) and a screw-retained crown was mounted 2–4 weeks later (Fig. 2H). The implant stability quotient (ISQ) was measured at each of these procedures using an Ostell® device (Ostell AB, Gothenburg, Sweden).

### Bone volume measurements and CBCT analyses

CBCT scans (Morita 3D Accuitomo F17, Japan) were taken before grafting (T0) and 6 months after grafting (T1), at 85 kVp, 9.5 mA with a field of view (FOV) of 6 cm × 6 cm (diameter × height), scanning time of 17.5 s, and a voxel size of 0.125 mm.

### Reconstruction of 3-dimensional models

The DICOM files of the images were then imported to Mimics program 19.0 (Materialize NV, Leuven, Belgium). The threshold of each case was selected manually, based on subjective evaluation of the apparent display of the residual jaw bone and the graft, this defined the boundary of the region of interest (ROI) of each case. The mask of the ROI at T0 was achieved and visualized in axial, sagittal, and coronal views. The 2D masks were then transformed into 3D models using the so-called “calculate 3D” function. The volume in cubic millimeters of the graft models was acquired automatically with a display of a color-coded 3D model.

The superimposition of the images at T0 and T1 was applied to the Standard Tessellation Language (STL) registration method [48]. Once the models were optimally superimposed, 3D models were reconstructed from the same region in the T0 and T1 images, specifying the augmented bone volumes (ROI).

### Processing bone biopsies

#### Micro-computed topography analyses

The bone biopsy specimens were maintained in 10% buffered formalin. Selected bone biopsies were scanned with the high-resolution μ-CT SkyScan1172® (SkyScan, Kontich, Belgium) with the following technical parameters: 100 mA and 100 kV power intensity, copper–alumina filter and 360^°^ rotation, and pixel size or resolution for acquisition and image reconstruction of 2.7 μm. Images from the scanning of biopsies were reconstructed by the software NRecon® (SkyScan) to obtain 2D and 3D images. CTvox (version 3.2; SkyScan) was employed to create 3D images for the biopsies. The analyzed histomorphometric parameters have been described previously [49]: bone volume (BV); tissue volume (TV); bone volumetric fraction (BV/TV); trabecular thickness (Tb.Th), the mean thickness of the trabeculae in the volume of interest (VOI); trabecular separation (Tb.Sp), the mean separation of the trabeculae in the VOI; structural model index (SMI), which gives information about the preponderance of trabecular morphology; degree of anisotropy (DA), which is the presence or absence of aligned trabeculae in a particular direction (1 is considered isotropic, > 1 is considered anisotropic); and fractal dimension (FD), which indicates the complexity of the specimen surface.

### Histological analyses

Fixed samples were decalcified in a pH 7.4 solution containing 4.13% EDTA/0.2% PFA in PBS for 96 h at 50 °C, using an automated microwave decalcifying apparatus (KOS Histostation; Milestone Med. Corp., USA). Samples were dehydrated in an ascending series of ethanol followed by butanol in an automated dehydration station (MicromMicrotech, Lyon, France). The samples were embedded in paraffin (Histowax; Histolab, Gothenburg, Sweden). Thin histological sections (3 μm thick) were made using a standard microtome (Leica RM2255; Leica Biosystems, Nanterre, France). The sections were stained by the Masson trichrome technique, which colors cell nuclei blue/black with hematoxylin, colors cytoplasm, muscle, and erythrocytes red using fuchsine, and colors collagen green using light green solution. Slides were scanned (NanoZoomer; Hamamatsu, Photonics, Hamamatsu City, Shizuoka, Japan) and observed virtually (NDP view; Hamamatsu). Histomorphometry of images was performed using ImageJ and the percentages of bone and bone marrow were calculated per area of explants. Four sections through each biopsy were analyzed and quantified.

### Statistical analysis

Bone width and volume are presented as means and confidence intervals. Confidence intervals were based on formulas assuming normal distributed data. The *p* value was calculated from a one-sample *t* test, with 0 as the hypothesized difference. *p* < 0.05 was considered statistically significant.

### Outcomes

The primary outcomes of the trial were safety and feasibility of the procedure, assessed 12 months after reconstruction. In order to evaluate safety, a system was established for reporting adverse events. With guidance from the European Medicines Agency, these events were further classified into serious adverse events or serious adverse reactions. Adverse events, local (e.g., infection or hematomas) or systemic (e.g., fever or allergic reaction), were to be managed according to the Guidelines for Good Clinical Practice from the International Conference on Harmonization and the German Verordnung über klinische Versuche mit Heilmitteln. The feasibility of the procedure was evaluated on the basis of two factors: surgical manipulation of the graft and the ability to install the implants as planned.

Secondary outcomes were osseointegration of the dental implant and function of the prosthetic restoration.

## Results

The final cell product consisted of fresh autologous cells (MSCs) expanded in vitro expressing the markers CD90, CD73, and CD105 and negative for CD14 and CD45, with a 90% viability rate. The product also showed strong expression of markers CD49d, CD73, CD90, and CD105; moderate expression of CD14 and CD106; and low expression of CD19, CD34, and CD45.

**Fig. 1:**
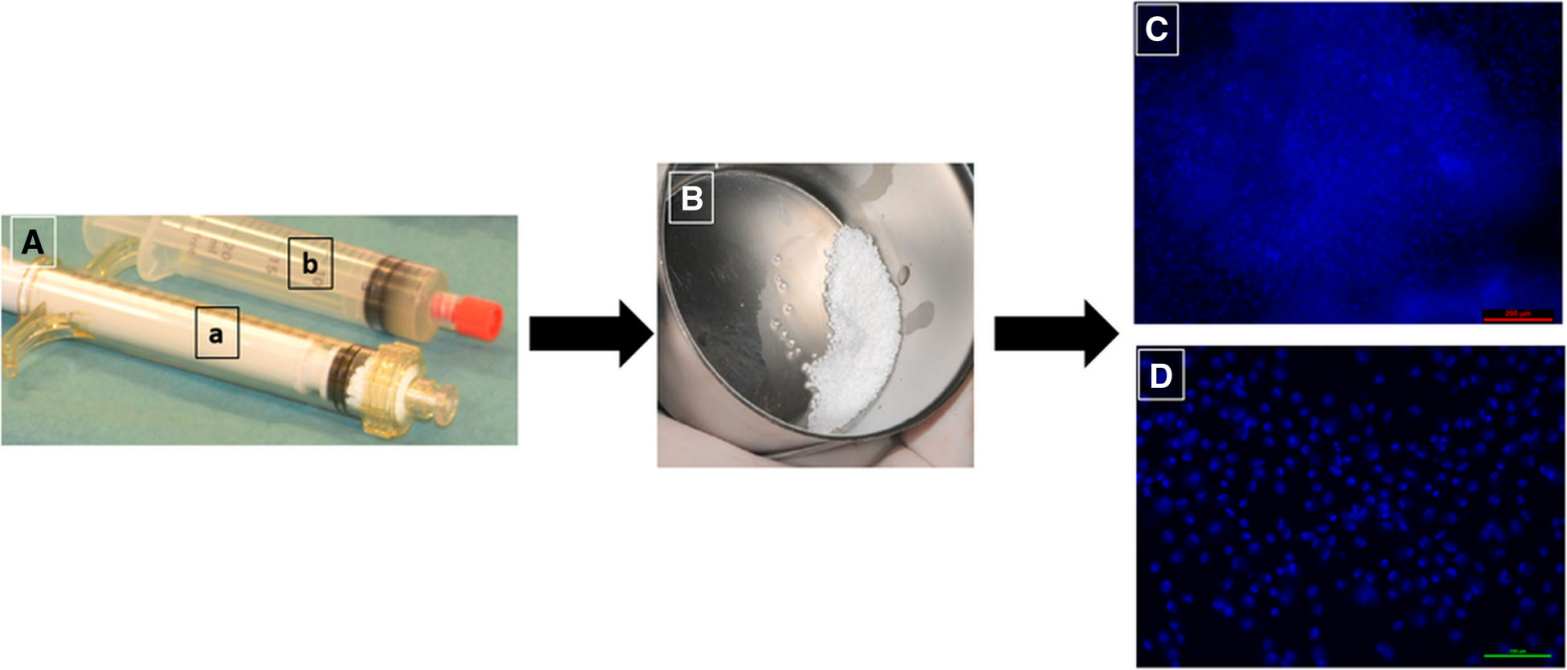
Cell attachment assay. **A** Syringes containing BCP granules (a) and MSCs (b). **B** Mixture of BCP and MSCs. **C**, **D** Cell attachment to biomaterial determined using DAPI staining after arrival at operating theater

The viability of the cells on arrival in the operating theater was 87–90% as demonstrated using Trypan blue assay and cell counting. The mixing was undertaken in theater by the surgeon, under aseptic surgical conditions (Fig. 1A, B). Cells were mixed and attached well to the BCP granules within 60 min (Fig. 1C, D).

Between June 2014 and December 2015, 13 patients aged 52–75 years (mean 65 years) were enrolled. For 11 of the 13 patients the expansions fulfilled the release criteria and cells could be delivered to the Department of Oral and Maxillofacial Surgery in Bergen. Two expansions were stopped at passage 0 because there were insufficient bone marrow cells in the starting material for expansion (Patients 5 and 10, Table 2).

**Table 2:** Expansion of cells derived from bone marrow of 13 patients

Patient number	BMSCs/μl BM number of MNCs	BMSCs/μl BM aspirate in passage 1	Overall harvest after culture passage 1
1	3.46E + 03	2.98E + 04	3.06E + 08
2	1.13E + 04	1.52E + 05	4.12E + 08
3	3.59E + 03	2.89E + 04	2.46E + 08
4	1.83E + 04	2.44E + 05	4.05E + 08
5	5.74E + 01	-^a^	-^a^
6	4.77E + 03	6.27E + 04	4.02E + 08
7	5.03E + 02	5.27E + 03	5.33E + 07
8	1.61E + 03	2.26E + 04	2.86E + 08
9	1,64E + 03	1.67E + 04	1.55E + 08
10	-^b^	-^a^	-^a^
11	6.54E + 03	7.67E + 04	2.42E + 08
12	2.70E + 03	2.85E + 04	2.69E + 08
13	3.63E + 03	2.79E + 04	2.34E + 08
Mean	4.84E + 03	6.32E + 04	2.74E + 08
SD	4.98E + 03	6.91E + 04	1.04E + 08

*BMSC* bone marrow-derived mesenchymal stromal cell, *BM* bone marrow, *MNC* mononuclear cell, *SD* standard deviation

^a^No colony-forming unit fibroblast CFU-F growth

^b^Insufficient cell count

All 11 patients had uneventful healing of the augmented area, without any local infection.

**Fig. 2:**
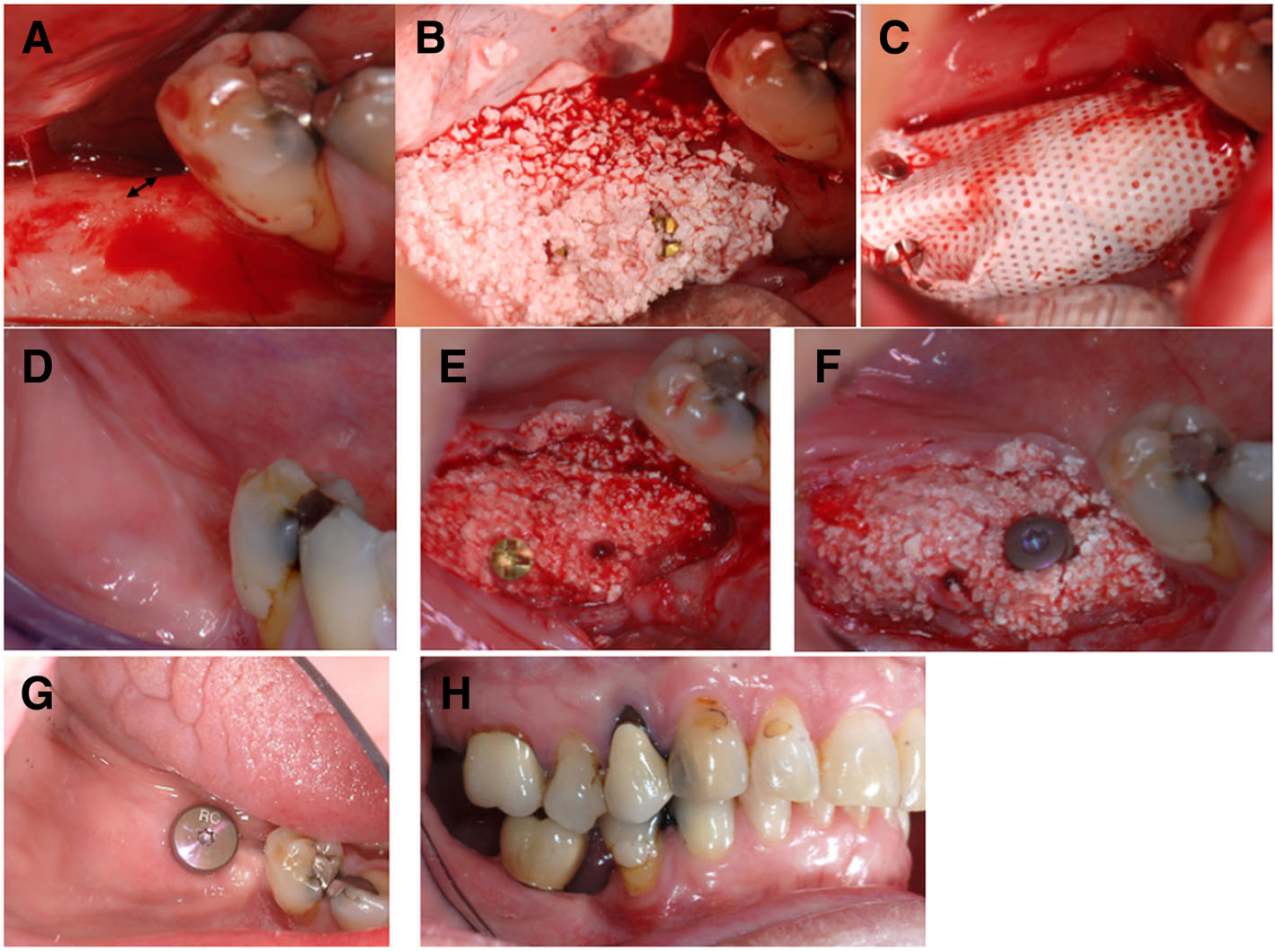
Clinical procedure. **a** Narrow alveolar ridge before augmentation (arrow). **b** Mixture of BCP and MSCs placed on alveolar ridge. **c** Membrane placed over transplanted graft. **d** Soft tissue healing after 5 months. **e** New alveolar ridge after 5 months of healing. **f** Core biopsy taken and dental implant installed on newly formed bone. **g** Eight months post augmentation and 2 months after implant installation. **h** Implant-supported crown in occlusion

No adverse events occurred during the trial period. Moreover, the soft tissues covering the augmented bone showed an increased area of keratinized gingiva, providing a healthy soft tissue profile (Fig. 2d). Finally, the amount of new bone was strongly influenced by the position of the membrane.

All 11 patients had successful ridge augmentation and an adequate amount of bone for dental implant installation (Table 3). In five patients the PTFE membrane became exposed and was removed uneventfully 7–8 weeks post augmentation.

**Table 3:** Clinical outcomes: demonstrates bone healing, increased bone width and volume

Patient number	Age (years)	Sex	Healing time (weeks)	Increase in width (mm)	Increase in volume (mm^3^)	Implant placement	Crown delivered	Patient satisfied
1	75	F	27	4.5	902.92	Yes	Yes	Yes
2	67	M	25	3.7	1047.15	Yes	Yes	Yes
3	55	F	26	3.9	1382.54	Yes	Yes	Yes
4	62	F	18	1.1	440.93	Yes	Yes	Yes
6	52	M	21	4.9	1469.53	Yes	Yes	Yes
7 left	69	M	31	4.6	432.7	Yes	Yes	Yes
7 right	69	M	31	4.9	1187.21	Yes	Yes	Yes
8	69	M	22	1.4	753.52	Yes	Yes	Yes
9	61	F	22	1.4	546.33	Yes	Yes	Yes
11	62	F	21	9.7	1188.47	Yes	Yes	Yes
12 left	65	F	20	2.7	954.98	Yes	Yes	Yes
12 right	65	F	20	3.4	418.36	Yes	Yes	Yes
13 left	69	F	22	3.7	553.56	Yes	Yes	Yes
13 right	69	F	22	6.8	1142.96	Yes	Yes	Yes

All patients received implants and prostheses

*F* female, *M* male

**Fig. 3:**
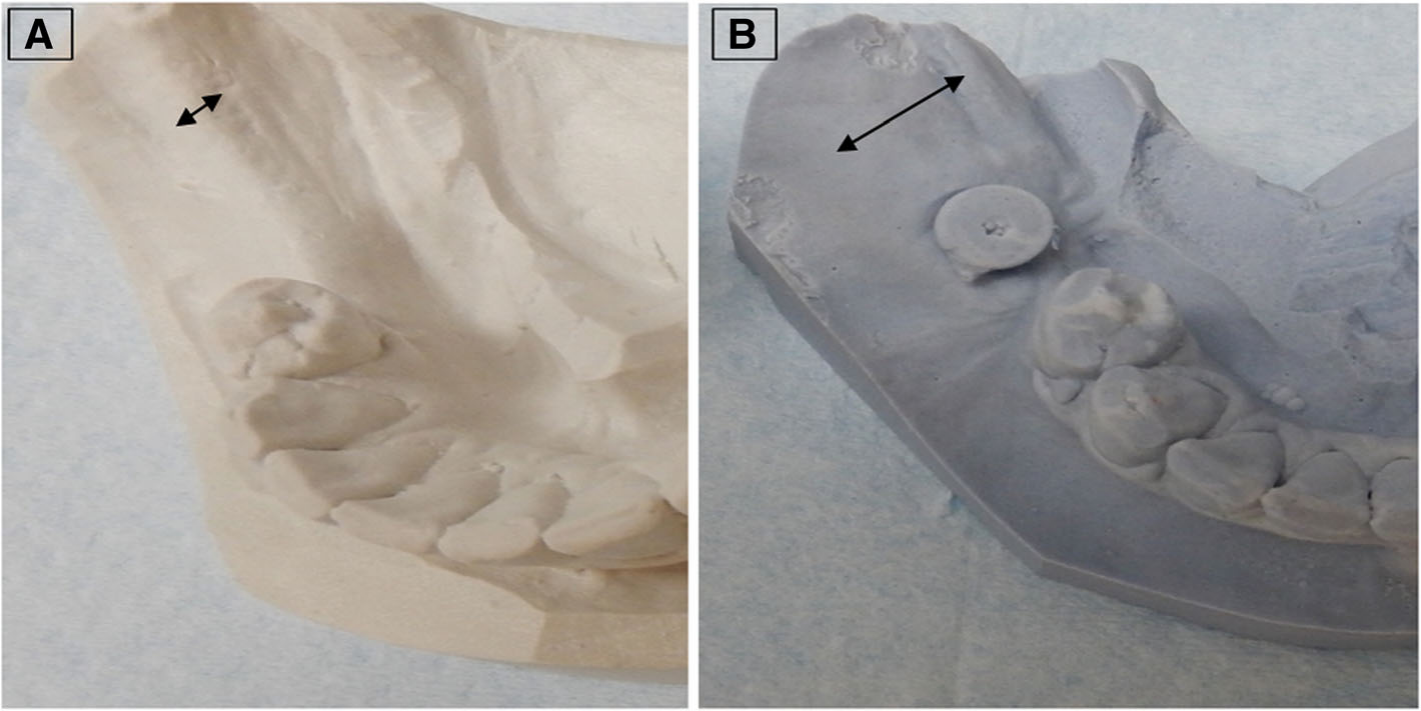
Cast of alveolar ridge. Before (**a**) and after (**b**) augmentation illustrating amount of bone reconstructed. Arrows indicate the width of the alveolar ridge

Casts of the alveolar ridge in each patient, X-ray scans, and clinical examinations demonstrated a significant increase of the total bone volume in all 11 patients after treatment (Fig. 3a, b).

**Fig. 4:**
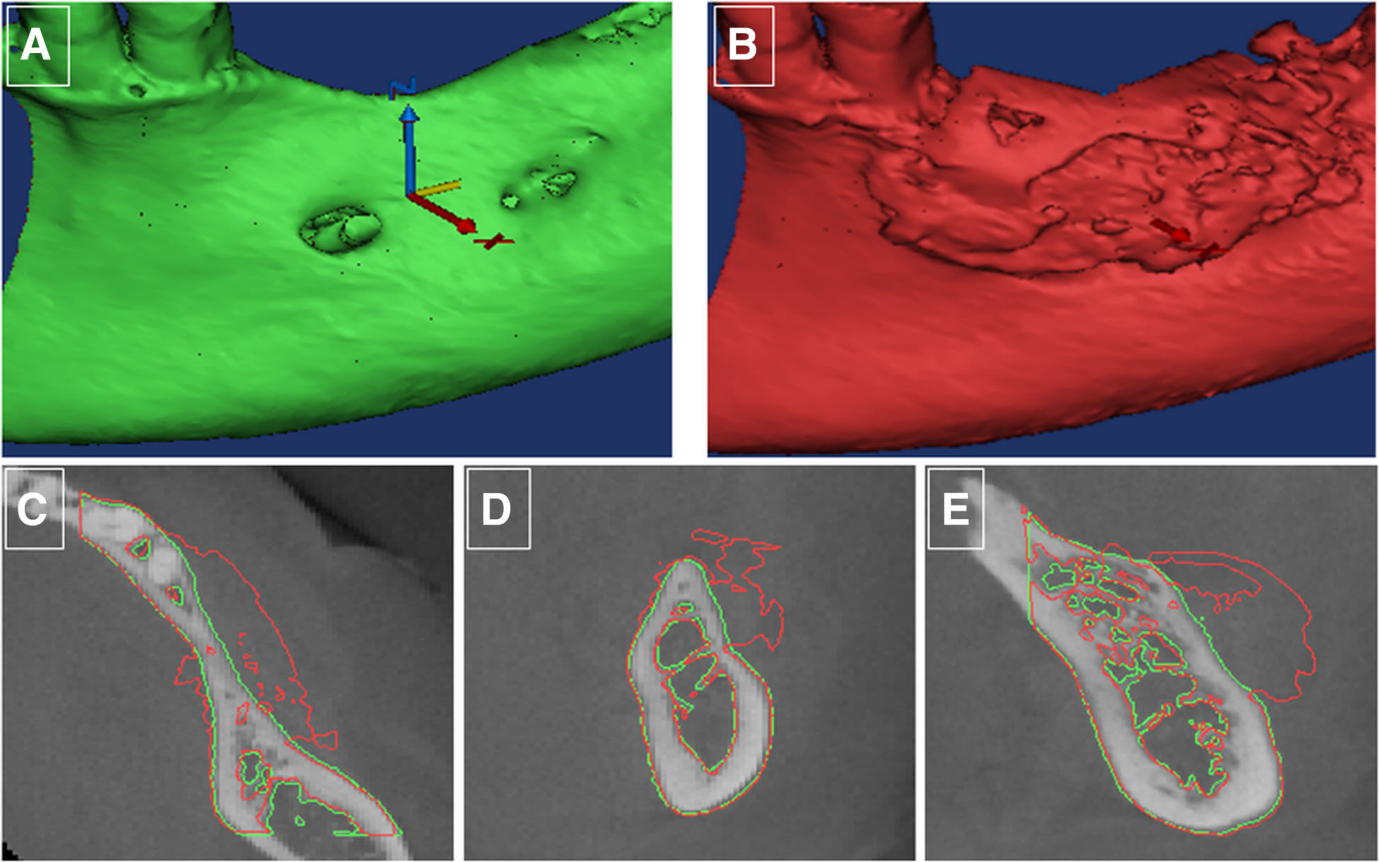
CBCT measurements. Overlapping of bone outline contours of superimposed models at T0 (before grafting, green) (**a**) and T1 (6 months after grafting, red) (**b**), achieved and viewed in axial (**c**), sagittal (**d**), and coronal (**e**) images of ridge before and after reconstruction

Linear measurements of the width and height were performed from all CBCT scans in iView software (version 2.2.0.3. J; Morita MFG Corporation). Grafted bone could easily be distinguished from residual bone by density and structure on the scans taken immediately after the grafting procedure. As these measurements are known to be operator dependent, the measurements were all done by one specialist in oral radiology (SS) [50, 51]. All patients had sufficient increase in alveolar width to have dental implants installed (Fig. 4 and Table 3). The average volume of bone increased by 887.23 ± 365.01 mm^3^ (Table 3). Both the increase in width of the alveolar ridge and the increase in volume of the alveolar ridge were statistically significant. The mean increase in bone width (*n* = 14) was 4.05 mm (95% CI 2.74, 5.36; *p* < 0.001) and the mean increase in volume (*n* = 14) was 887.23 mm^3^ (95% CI 676, 1097.98; *p* < 0.001).

**Fig. 5:**
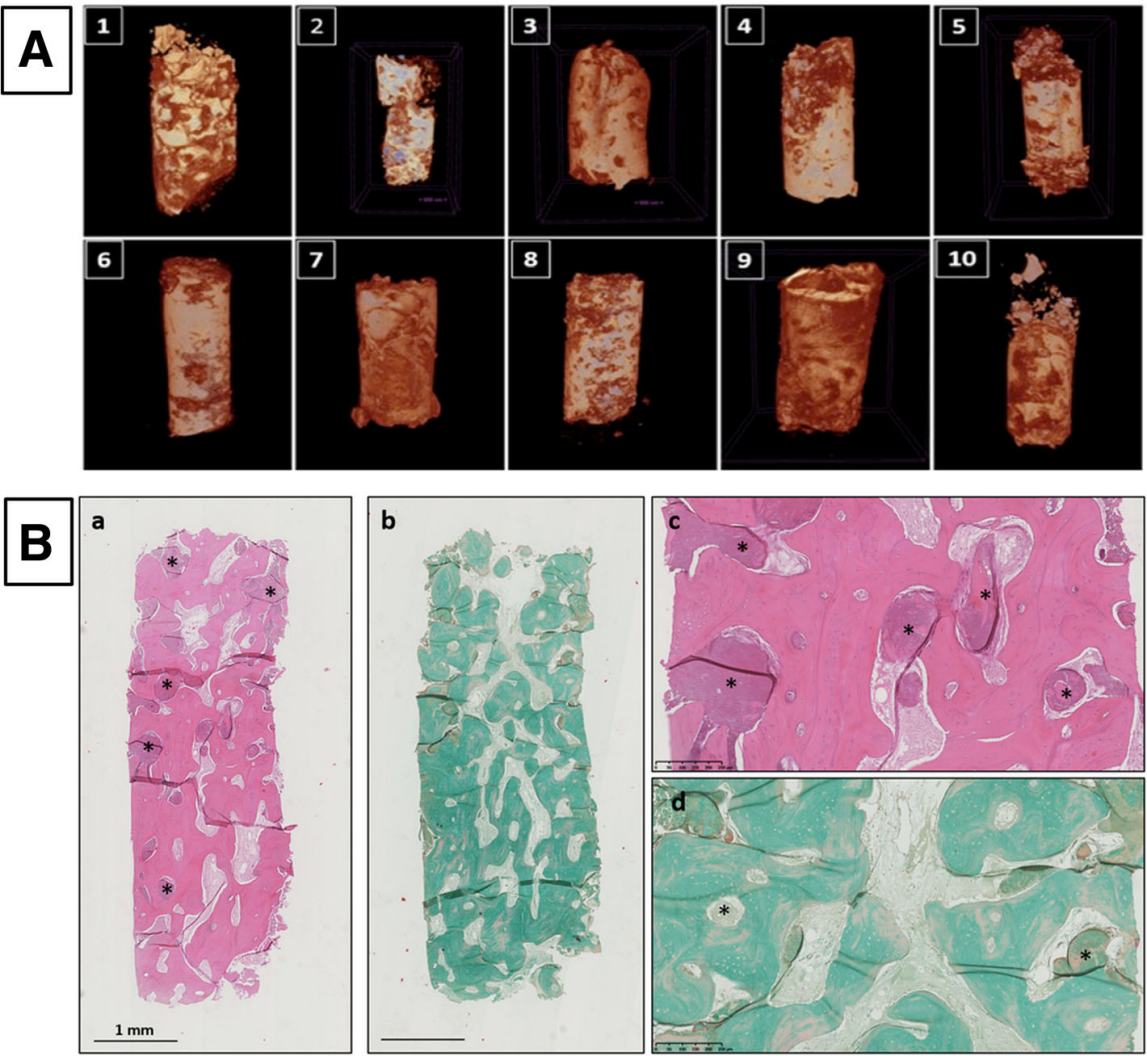
μ-CT and histological analyses. **A** μ-CT images of biopsies from Patients 1–10. **B** Histology of core biopsies from patients. Note abundant lamellar bone with entrapped osteocytes in extracellular matrix at high magnification around remaining BCP particles (*). a, c Hematoxylin and eosin staining, b, d Masson trichrome staining. Magnification ×1.25 and ×10

Formation of mineralized tissues was evaluated by μ-CT and histology from the biopsies taken during implant installation. From the μ-CT scan datasets, 3D models were built for visualization (Fig. 5A). It was possible to identify accurately the newly formed bone from the BCP granules (based on histogram calculations) when the raw data-reconstructed cross-sections were turned into images.

Histological analysis revealed that BCP granules were well integrated with deposition of newly formed bone tissue on the surface of the particles with osteoblast lining cells and subsequent deposition of lamellar bone tissue (Fig. 5B). The BCP granules demonstrated continuous degradation and dissolution, with the presence of multinucleated cells, probably osteoclasts, as well as macrophage CD68^+^ cells on the surface of the particles.

Table 4 presents the mean values for each analyzed variable obtained by μ-CT analyses in relation to the microstructural properties of the biopsies.

**Table 4:** Mean values for each analyzed variable in relation to microstructural properties of the biopsies

Patient	TV (mm^3^)	BV (mm^3^)	BV/TV (%)	Th.Tb (mm)	Tb.Sp (mm)	SMI	DA	FD
1	5.187	1.2	23.131	0.023	0.131	0.542	1.153	2.63
2	5.436	0.961	17.677	0.046	0.251	0.277	1.29	2.485
3	4.717	0.495	10.501	0.004	0.359	0.742	1.367	2.256
4	5.333	0.963	18.055	0.039	0.288	0.354	1.256	2.467
5	5.358	0.791	14.762	0.033	0.279	0.529	1.107	2.422
7	4.933	0.741	15.022	0.031	0.239	0.215	1.410	2.46
8	5.546	0.881	15.891	0.045	0.255	0.812	1.549	2.46
9	4.413	0.568	12.867	0.032	0.25	0.437	1.333	2.390
11	5.064	1.106	21.844	0.051	0.180	0.740	1.144	2.542
12	5.488	0.567	10.317	0.037	0.246	0.609	1.333	2.433

In Patient 13, the biopsy disintegrated during transport and could not be measured. However, all dental implants have osseointegrated and are still in successful clinical function

*TV* tissue volume, *BV* bone volume, *BV/TV* bone volumetric fraction, *Tb.Sp* trabecular separation, *Th.Tb* Trabercular thickness, *SMI* structural model index, *DA* degree of anisotropy, *FD* fractal dimension

**Fig. 6:**
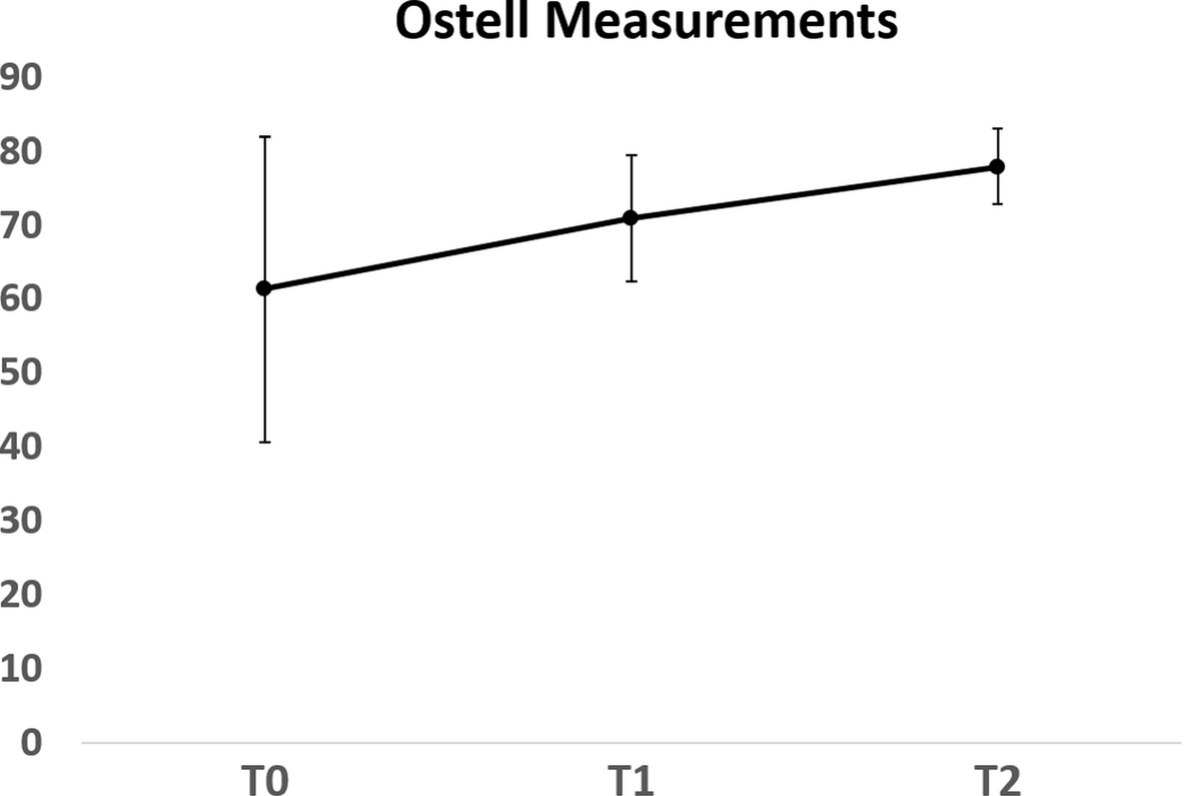
Ostell measurements. Implant installation (T0), at loading (T1), and at 18 months follow-up (T2). Data presented as mean ± SD showing increased implant stability after loading

All patients were satisfied with the esthetic and functional outcomes and no adverse events were reported or observed. There were no postoperative infections in any of the transplants or at the donor site. One patient reported moderate levels of pain after augmentation and after the exposed membrane had to be removed. The other patients reported only minor pain postoperatively. All patients were satisfied with the clinical outcome of the augmentation procedure and with their new teeth. All patients said they would recommend this procedure to others with a similar clinical condition. Ostell values increased for all patients during the first 12 months after installation of the dental implants (Fig. 6).

## Discussion

Successful augmentation of alveolar bone was observed in all study participants in this clinical trial of a novel protocol using bone marrow-derived MSCs. The site selected for bone augmentation was the posterior mandibular ridge. This is one of the most challenging sites for reconstruction, because of the relatively limited blood supply [52, 53], nonsterile environment [54], and oral functions such as chewing, speaking, and swallowing, which interfere with the stability of the graft. Despite these obstacles and the use of granules as scaffolding, we succeeded in inducing the formation of significant new bone and increasing the volume of the alveolar ridge.

Horizontal bone augmentation of the alveolar ridge is considered to be predictable, whereas vertical augmentation is not [55, 56]. Major drawbacks in relation to the bone graft treatment are donor side morbidity, limited amount of bone to be harvested, and unpredictable resorption of the graft [7, 57–62]. Using the stem cell/biomaterial approach in the present trial promoted both horizontal and vertical augmentation [56]. The donor site morbidity reported by the patients was minimal. The novelty of this approach was related to the development of an appropriate protocol to produce clinical-grade cells that could be used successfully for bone regeneration. The MSCs were expanded using no osteogenic factors, and no osteogenic factors were used in the clinical procedure [63–65], as growth factors may have different effects on different tissue [66] and also increase the cost of producing the cells.

In preclinical studies, MSCs were expanded and produced by the manufacturing center according to the protocol used in this clinical trial. Cells were shipped within 24 h and applied fresh in different animal models to demonstrate the formation of new bone in combination with the BCP biomaterial [39, 67]: the biomaterial alone fails to bridge bone defects in critical size calvarial defects in nude mice while full bridging was achieved with MSC/BCP combinations [39]. However, formation of bone seems to be dependent on a critical number of cells or a critical cell-to-biomaterial ratio. The number of cells and the cell-to-biomaterial BCP ratio used in this clinical study were adapted from the preclinical findings, where 20 × 10^6^ MSCs were mixed with 1 cm^3^ BCP [39]. We believe that the intrinsic capacity of MSCs to form bone makes the trial reproducible and safer, because the cells were not manipulated. However, a positive effect on osteogenic “predifferentiation” of MSCs using PL as a supplement during the isolation and expansion phases cannot be excluded, although this has been a somewhat controversial topic [68, 69]. In this clinical trial, PL was produced from up to 80 individual donors: as shown in a recent study, this minimizes variations in the content of growth factors, chemokines, and cytokines [44] and ensures stable conditions for the ex-vivo expansion of MSCs.

Two of the patients had insufficient cell expansion in vitro, perhaps due to the variable content of MSCs (CFU-F) in bone marrow aspirates from different individuals [65]. This variability may be a limiting step in the procedure, but may be overcome by increasing the number of cells harvested or by developing methods for identifying the relevant cells prior to initiating culture.

There are few published papers on mandibular and maxillary defect reconstruction using bone marrow or adipose-derived stem cells [33, 70–77], many of which are case reports [70, 74, 75, 77]. The published studies vary in cell source, defect site, scaffold material, cell number, use of growth factors, and membrane or hardware [33, 70, 74–79]. However, the present data generated by treating 11 cases differ from these earlier reports as no growth factor or stimulants were used on the cells prior to implantation. Furthermore, the posterior mandibular region (i.e., distal to the canine) in all patients was selected as an inclusion criterion, as the bone healing is dependent on the location of the defected bone. Although the membrane was the determinant of augmentation volume, it complicated the surgical procedure and postoperative healing procedure. The high-density membrane is microporous, impervious to bacteria while still allowing diffusion of gases and small molecules, but probably inhibits vascularization from the periosteum, limiting the blood supply to the graft. The granules that remained outside the compartment made by the membrane did not induce bone formation, indicating the importance of using an appropriate membrane. Further supporting the importance of the membrane in bone formation, a study by Meijer et al. [76] using no membrane and grafts of bone marrow MSCs grown for 7 days in osteogenic medium and loaded with ceramic bone substitutes did not succeed in inducing bone formation.

In a randomized, controlled trial reported recently, osseous defects generated after tooth extraction were treated successfully with bone marrow-derived cells loaded on gelatin sponge. They showed accelerated healing after 6 weeks, but no significant difference after 12 weeks compared to no cells applied to the defect [33]. However, it is well known that extraction sockets heal without intervention [58, 80].

In the present study, the volumetric measurement on CBCT images was a visual protocol for assessing the outcome of grafting. The volumetric changes to the bone were achieved at T0 and T1. The objective measurement on CBCT images was performed to confirm the clinically observed volumetric changes in the graft [81–83]. This methodology has also been used in follow-up after grafting procedures in alveolar cleft patients [84–86]. Further, the biopsy specimens taken 4–6 months after augmentation showed significant new bone formation, with abundant blood supply and without inflammatory cells. The BCP scaffold was still visible in the histological samples as the reported resorption time is up to 2 years [38]. The scaffold material provides the extracellular microenvironment for support and stimulation of the cells, and also acts as the delivery system for the cells [18]. Although no direct evidence is provided relative to the source of the cells that produced the regenerated tissue (i.e., labeling of the cells), the assumption can be made that the transplanted cells at least partly contributed to bone regeneration, because the bone core specimen was taken from the central region of the defect and graft site.

Normally, there is a gradual resorption of keratinized mucosa simultaneously with bone resorption and this resorbed keratinized mucosa is known to not regenerate [87, 88]. The presence of keratinized mucosa of at least 1–2 mm around an implant is beneficial in decreasing plaque accumulation, tissue inflammation, and attachment loss [87, 89]. In our patients, an unexpected benefit of the augmentation procedure was an increase in the width of keratinized mucosa (Fig. 2d, g). It therefore appears that the cells used to regenerate bone also have a positive effect on neighboring soft tissues and contribute to wound healing, even when covered by a membrane. MSCs have demonstrated a beneficial effect on wound healing [90, 91]. This observation warrants further investigation. However, MSCs have demonstrated a beneficial effect on wound healing, which appears to be mediated by paracrine signaling [91]. The role of paracrine factors produced by stem cells in tissue regeneration and healing has been investigated and reports showed that angiogenesis and osteogenesis were promoted in response to the paracrine effect of stem cells [65, 90]. This paracrine effect is exerted through cytokines and chemokines such as insulin-like growth factor (IGF)-1, vascular endothelial growth factor (VEGF), and transforming growth factor (TGF)-β1. These growth factors were found to enhance cell proliferation, mobilization, angiogenesis, and expression of osteogenic markers such as alkaline phosphatase, collagen type I, and Runx2 genes [92]. Furthermore, these factors recruit endogenous stem cells to the grafted site [90, 92].

Because of the small cohort and follow-up time (now up to 3 years), the promising results of this study should be interpreted with caution. In order to validate this treatment protocol for application in a standard clinical setting, further study is warranted, with a larger study cohort and a longer follow-up period. Nevertheless, the results of this study are promising and could lead to the development of new strategies for regenerative medicine and therapeutic interventions, and thus have a direct and positive impact on large groups of patients.

## Conclusions

The results of this novel clinical study in human subjects show that clinical reconstruction of the alveolar ridge using autologous MSCs and BCP is feasible, safe, and predictable. All sites were successfully augmented; all dental implants osseointegrated and were restored with screw-retained dental crowns as planned. Hence, this novel augmentation procedure warrants further investigation and may form the basis of a valid treatment protocol, challenging the current gold standard.

## Abbreviations

BCP: Biphasic calcium phosphate
BM: Bone marrow
BV: Bone volume
BV/TV: Bone volumetric fraction
CBCT: Cone beam computer tomography
μ-CT: Micro-computed tomography
DA: Degree of anisotropy
FD: Fractal dimension
FOV: Field of view
HA: Hydroxyapatite
IGF-1: Insulin-like growth factor
ISQ: Implant stability quotient
αMEM: Minimal Essential Medium alpha modification
MSC: Mesenchymal stromal cell
PL: Platelet lysate
PTFE: Polytetrafluoroethylene
ROI: Region of interest
SMI: Structural model index
STL: Standard Tessellation Language
Tb.Sp: Trabecular separation
Tb.Th: Trabecular thickness
β-TCP: Beta tricalcium phosphate
TGF: Transforming growth factor
TV: Tissue volume
VEGF: Vascular endothelial growth factor
VOI: Mean thickness of the trabeculae in the volume of interest
WBC: White blood cell
